# Closed‐loop systems in Type 1 diabetes and antitumor necrosis factor alpha therapy: Are currently available algorithms prepared for sudden changes in insulin sensitivity?

**DOI:** 10.1111/1753-0407.13515

**Published:** 2023-12-19

**Authors:** Lía Nattero‐Chávez, Jorge Bondía, Héctor F. Escobar‐Morreale, Manuel Luque‐Ramírez

**Affiliations:** ^1^ Department of Endocrinology and Nutrition Hospital Universitario Ramón y Cajal Madrid Spain; ^2^ Diabetes, Obesity and Human Reproduction Research Group Universidad de Alcalá, Instituto Ramón y Cajal de Investigación Sanitaria (IRYCIS) Madrid Spain; ^3^ Centro de Investigación Biomédica en Red de Diabetes y Enfermedades Metabólicas Asociadas Instituto de Salud Carlos III Madrid Spain; ^4^ Instituto Universitario de Automática e Informática Industrial Universitat Politécnica de Valencia Madrid Spain

Dear Editor,

Automated insulin delivery by closed‐loop systems (CLS) improves metabolic control in Type 1 diabetes (T1D), providing a flexible platform capable of matching their changing insulin requirements.^1^ We hereby report the case of a 24‐year‐old woman being adequately controlled by a CLS, whose T1D became unstable following a tumor necrosis factor alpha inhibitor (iTNF‐α). After onset of diabetes at the age of 12, her metabolic control worsened progressively, with mean glycated hemoglobin (HbA_1c_) reaching 8.4% despite on multiple daily insulin injections supported by a carbohydrate counting plan. Accordingly, the patient started MiniMed 780G (Medtronic Northridge, California) in April 2022. After 1 month of CLS, HbA_1c_ decreased to 7.3%. She progressively developed severe hidradenitis suppurativa (HS). Having failed conventional management, her dermatologist prescribed adalimumab, starting on September 14, 2023. The patient experienced substantial glycemic variability (GV), increasing time below range (TBR) within 2 weeks of starting adalimumab. Data from continuous glucose monitoring confirmed the increase in TBR from 2% to 7% and GV from 31% to 39% when comparing the 2 weeks with adalimumab with the 2 weeks preceding its start. The risk of hypoglycemia showed an overlap of several automatic self‐correcting insulin boluses, despite adequate suspension of the automatic basal rate and late postprandial hypoglycemia despite basal insulin delivery being discontinued (Figure 1). Suspecting an increase in insulin sensitivity due adalimumab, the configuration of CLS was changed, increasing the target to 120 mg/dL, active insulin duration to 4 h, and canceling the corrective boluses. Finally, we recommended her dermatologist to find a pharmacological alternative for HS.

**FIGURE 1:**
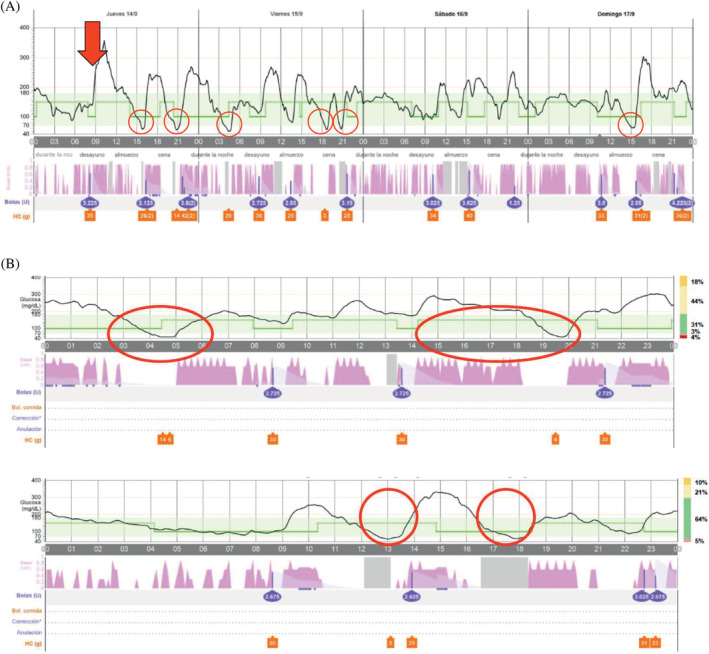
(A) First 4 days following start of treatment with adalimumab. Arrow indicates first dose of drug administration; circles indicate Level 2 of hypoglycemia episodes. (B) Continuous glucose monitoring data show Level 2 hypoglycemic events related to accumulation of automatic correction doses despite automatic basal suspension or despite manual pump suspension (gray area).

Cell immunity and proinflammatory cytokines including TNF‐α are involved in the pathogenic process of HS.^2^, ^3^ Data on sex hormones suggest that androgen excess may favor HS development in female patients. In conceptual agreement, the presence of functional hyperandrogenism in one out of every four female patients with T1D may underline the high frequency of newly diagnosed HS among these women.^4^

Hypoglycemia induced by iTNF‐α is related to reduction in inflammation‐induced insulin resistance. TNF‐α induces insulin resistance by serine/threonine phosphorylation of the insulin receptor 1 substrate, interferes with normal phosphorylation of tyrosine, and impairs signal transduction of insulin resulting in insulin resistance through increasing the activities of the NF‐κB transcriptional factor, protein kinase C, amino terminal kinase, and inhibitor kinase.^5^ We hypothesized that adalimumab led to a sudden increase in insulin sensitivity in our patient, not adequately compensated by the CLS, increasing the risk of hypoglycemia.

The main concept underlying learning is “run‐to‐run control”, widely applied in batch processes, where adjustments are made after each batch. In the context of a CLS, a “batch” is considered as a given past‐time window, where specific glycemic control features are measured making parameters adjustments accordingly, usually with constraints for the sake of safety. This requires batch‐to‐batch repeatability for convergence, as high variability is a challenge for self‐learning. This clinical case illustrates the limitation of current CLS in adapting to sudden changes in insulin sensitivity. We must advise patients with a CLS and iTNF‐α to make preventive adjustments to the algorithm configuration to avoid hypoglycemic events.

## FUNDING INFORMATION

This report is not funded.

## DISCLOSURE

None of the authors have any competing financial interest to disclose.
